# High prevalence of vitamin D deficiency among children aged 1 month to 16 years in Hangzhou, China

**DOI:** 10.1186/1471-2458-12-126

**Published:** 2012-02-14

**Authors:** Zhiwei Zhu, Jianying Zhan, Jie Shao, Weijun Chen, Liqin Chen, Wenhao Li, Chai Ji, Zhengyan Zhao

**Affiliations:** 1 Department of Child Health Care, Children's Hospital Affiliated to Zhejiang University School of Medicine, Hangzhou, China; 2 Department of Central laboratory, Children's Hospital Affiliated to Zhejiang University School of Medicine, Hangzhou, China

## Abstract

**Background:**

Recent studies have suggested that vitamin D deficiency in children is widespread. But the vitamin D status of Chinese children is seldom investigated. The objective of the present study was to survey the serum levels of 25-hydroxyvitamin D [25(OH)D] in more than 6,000 children aged 1 month to 16 years in Hangzhou (latitude: 30°N), the capital of Zhejiang Province, southeast China.

**Methods:**

The children aged 1 month to 16 years who came to the child health care department of our hospital, the children's hospital affiliated to Zhejiang university school of medicine, for health examination were taken blood for 25(OH) D measurement. Serum 25(OH) D levels were determined by direct enzyme-linked immunosorbent assay and categorized as < 25, < 50, and < 75 nmol/L.

**Results:**

A total of 6,008 children aged 1 month to 16 years participated in this cross-sectional study. All the subjects were divided into subgroups according to their age: 0-1y, 2-5y, 6-11y and 12-16y representing infancy, preschool, school age and adolescence stages respectively. The highest mean level of serum 25(OH)D was found in the 0-1y stage (99 nmol/L) and the lowest one was found in 12-16y stage (52 nmol/L). Accordingly, the prevalence of serum 25(OH)D levels of < 75 nmol/L and < 50 nmol/L were at the lowest among infants (33.6% and 5.4% respectively) and rose to the highest among adolescents (89.6% and 46.4% respectively). The mean levels of serum 25(OH)D and the prevalence of vitamin D deficiency changed according to seasons. In winter and spring, more than 50% of school age children and adolescents had a 25(OH)D level at < 50 nmol/L. If the threshold is changed to < 75 nmol/L, all of the adolescents (100%) had low 25(OH)D levels in winter and 93.7% school age children as well.

**Conclusions:**

The prevalence of vitamin D deficiency and insufficiency among children in Hangzhou Zhejiang province is high, especially among children aged 6-16 years. We suggest that the recommendation for vitamin D supplementation in Chinese children should be extended to adolescence.

## Background

Vitamin D deficiency causes rickets in infants and young children. However, rickets can be considered only the tip of the vitamin D deficiency iceberg. The vitamin D receptor is present in the small intestine, colon, osteoblasts, activated T and B lymphocytes, β islet cells, mononuclear cells and most organs in the body including the brain, heart, skin, gonads, prostate, and breast [1]. Many studies over the last 2 decades have suggested important role vitamin D plays in decreasing the risk of many chronic diseases, including cancers[2,3], autoimmune diseases[4,5], infectious diseases[6-8], and cardiovascular diseases[9].

Although there is no consensus on optimal levels of 25-hydroxyvitamin D as measured in serum, vitamin D deficiency is defined by most experts as a 25-hydroxyvitamin D level of less than 50 nmol per liter [10]. After reviewed studies on the thresholds for serum 25(OH)D concentrations in relation to bone mineral density (BMD), lower extremity function, dental health, and risk of falls, fractures, and colorectal cancer, Heike A Bischoff-Ferrari found that, the most advantageous serum concentrations of 25(OH)D begin at 75 nmol/L, and the best are between 90 and 100 nmol/L[11]. In children, the optimal level of serum 25(OH)D for general health is unknown and even more controversial than in adults, because there are fewer outcome data[12,13]. It has been proposed that the minimal optimal circulating vitamin D level should be increased from 50 nmol/L to 75 nmol/L[14,15], because there is evidence that biochemical sequelae of vitamin D deficiency may manifest at cutoff levels of 75 nmol/L[11,16]. And the level of 25-hydroxyvitamin D between 50 and 75 nmol/L can be considered as a relative insufficiency of vitamin D in children.

According to such definitions, the prevalence of vitamin D insufficiency was higher among American children aged 6-11 years (73%) compared with children aged 1-5 years (63%); girls (71%) compared with boys (67%); and non-Hispanic black (92%) and Hispanic (80%) children compared with non-Hispanic white children (59%)[17]. In studies in Australia, Turkey, India, and Lebanon, 30-50% of children and adults had 25(OH)D levels under 50 nmol per liter[18-20]. But the vitamin D status of Chinese children is seldom investigated, especially in southeast China. Hangzhou, the capital of Zhejiang province is located in the southeast of China at northern latitude 30°. In the present study, we analyzed serum 25(OH)D levels of more than 6,000 Chinese children aged 1 month to 16 years in Hangzhou. These data would be important in drawing the policy to prevent vitamin D deficiency and improving the vitamin D status in Zhejiang province as well as all over China.

## Methods

### Survey and sample

Data collection took place between March 2008 and February 2011, throughout the year, during winter (December through February), spring (March through May), summer (June through August), and autumn (September through November). The children aged 1 month to 16 years who came to the child health care department of our hospital, the Children's Hospital affiliated to Zhejiang university school of medicine, for health examination were taken blood for 25(OH)D measurement. Children were excluded if they were diagnosed as rickets or hypocalcemia or had abnormal liver or renal function that might affect hydroxylation of vitamin D, calcium and phosphorus metabolism. Informed consent was obtained from all parents. This study was approved by the Children's Hospital institutional review board.

### 25-hydroxyvitamin D measurement

Circulating levels of 25-hydroxyvitamin D [25(OH)D] are considered to be the most reliable measure of overall vitamin D status[1]. In the present study, the serum 25(OH)D levels were measured by a direct enzyme-linked immunosorbent assay with the 25-Hydroxy Vitamin D Direct EIA kit purchased from Immunodiagnostic Systems Ltd. (Fountain Hills, Arizona, USA). This assay has been shown to reliably measure both 25-hydroxyvitamin D isoforms (D_2 _and D_3_) [21].

### Statistical analyses

Mean serum levels of 25(OH)D were computed and campared between groups by using the 2-tailed *t *test or analysis of variance where appropriate. Differences were considered significant at *P *< 0.05. For purposes of analyses, the prevalence of vitamin D deficiency was based on the proposed definition of < 50 nmol/L, and cutoff values of < 25 nmol/L and < 75 nmol/L were used to describe overt vitamin D deficiency and insufficiency, respectively. Statistical analyses were performed by using the program package SPSS 13.0.

## Results

### Participant characteristics

A total of 6,008 children aged 1 month to 16 years participated in this cross-sectional study. All the participants were divided into four stages according to their ages: 0-1y stage, 2-5y stage, 6-11y stage and 12-16y stage representing infancy, preschool, school age and adolescence stages respectively. The characteristics of the participants were shown in Table 1.

**Table 1:** Characteristics of study sample and distribution of serum 25 (OH) D

	Mean+/- SD(nmol/L)	*P*value	< 25 nmol/L,% (95%CI)	< 50 nmol/L,% (95%CI)	< 75 nmol/L,% (95%CI)
**0-1y stage (infancy)**					

**Total (n = 2116)**	98.7 ± 47.1		0.4 (NC)	5.4 (4.4-6.4)	33.6 (31.6-35.6)

**Gender**		0.763			

**Male (n = 1221)**	98.5 ± 47.0		0.4 (NC)	5.3 (4.0-6.6)	34.3 (31.6-37.0)

**Female (n = 895)**	99.1 ± 47.2		0.3 (NC)	5.5 (4.0-7.0)	32.7 (29.6-35.8)

**2-5y stage (preschool)**					

**Total (n = 2269)**	69.6 ± 30.4		1.1 (NC)	21.9 (20.2-23.6)	68.6 (66.7-70.5)

**Gender**		0.108			

**Male (n = 1454)**	70.4 ± 31.4		1.2 (NC)	20.8 (18.7-22.9)	67.7 (65.3-70.1)

**Female (n = 815)**	68.2 ± 28.6		1.1 (NC)	24.0 (21-27)	70.1 (67.0-73.2)

**6-11y stage (school age)**					

**Total (n = 1440)**	56.1 ± 19.9		2.0 (NC)	40.4 (37.9-42.9)	88.3 (86.6-90.0)

**Gender**		0.969			

**Male (n = 1019)**	56.1 ± 18.7		1.6 (NC)	39.1 (36.1-42.1)	88.8 (86.9-90.7)

**Female (n = 421)**	56.1 ± 22.6		3.1 (NC)	43.7 (39.0-48.4)	86.9 (83.7-90.1)

**12-16y stage(adolescence)**					

**Total (n = 183)**	52.1 ± 17.0		3.3 (NC)	46.4 (39.2-53.6)	89.6 (85.2-94.0)

**Gender**		0.051			

**Male (n = 135)**	53.5 ± 17.0		2.2 (NC)	44.4 (36-52.8)	88.1 (82.6-93.6)

**Female (n = 48)**	48.0 ± 16.6		6.3 (NC)	52.1 (38-66.2)	93.8 (87.6-100)

CI indicates confidence intervals and NC indicates not calculable (n < 30 observations).

### Serum 25-hydroxyvitamin D levels and prevalence of low vitamin D status

The mean serum 25(OH)D levels of those children aged 1 month to 16 years decreased while their ages increased. The mean serum 25(OH)D level was the highest (98.7 nmol/L) among infancy and decreased sharply among preschoolers (69.6 nmol/L) and school children (56.1 nmol/L), then reached the lowest among adolescents (52.1 nmol/L) (Table 1).

Accordingly the prevalence of 25(OH)D levels at < 75 nmol/L, < 50 nmol/L and < 25 nmol/L increased while the children got older. More than 1 of every 5 preschoolers and over 40% of school children and adolescents (40.4% and 46.4%, respectively) had 25(OH)D levels of < 50 nmol/L. If the threshold is changed to < 75 nmol/L, almost all children aged 6-16 years had low levels (88.3% among school children and 89.6% among adolescents) and most children aged 2-5 years did as well (68.6%) (Table 1).

No significant differences were found between the mean serum 25(OH)D level of boys and that of girls at any stage. But the mean serum 25(OH)D level among adolescent girls was lower than that of boys, P = 0.05 (Table 1).

### Serum 25-hydroxyvitamin D levels changed in different seasons

**Figure 1:**
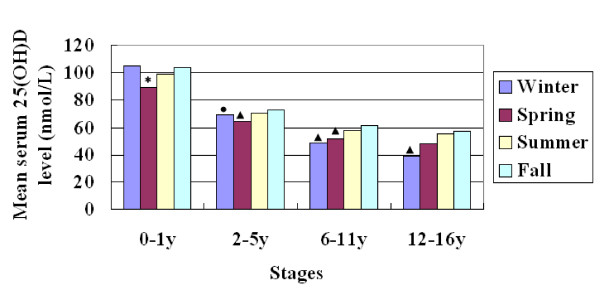
**The mean serum 25(OH) D level changed according to seasons at each stage**. *, *P *< 0.001 when compared with all other seasons at the same stage. ●, *P *< 0.05 when compared with spring and autumn at the same stage, ▲, *P *< 0.001 when compared with summer and autumn at the same stage.

**Figure 2:**
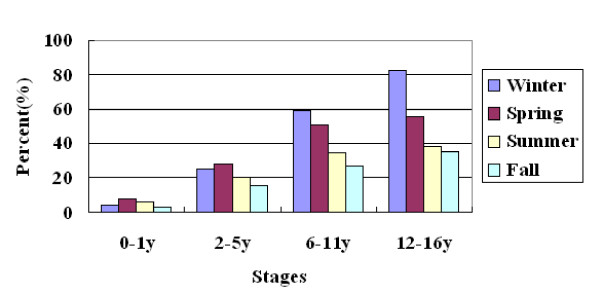
**Prevalence of 25(OH)D level at < 50 nmol/L according to seasons at each stage**.

**Figure 3:**
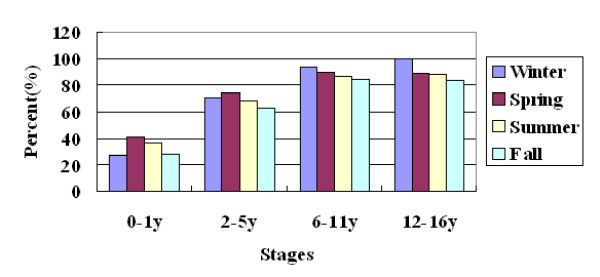
**Prevalence of 25(OH)D level at < 75 nmol/L according to seasons at each stage**.

The mean levels of serum 25(OH)D changed according to seasons. Among school age children and adolescents, the levels of 25(OH)D gradually increased in the order of winter, spring, summer and autumn (from 49 nmol/L to 61.1 nmol/L among school age children and from 38.6 nmol/L to 56.9 nmol/L among adolescents) (Figure 1). Accordingly, the prevalence of 25(OH)D levels at < 75 nmol/L and < 50 nmol/L decreased gradually from winter to autumn among those older children. In winter and spring, more than 50% percent of school age children and adolescents had a 25(OH)D level at < 50 nmol/L (Figure 2). If the threshold is changed to < 75 nmol/L, all of the adolescents (100%) had low 25(OH)D levels in winter and 93.7% school age children as well. Even in autumn, there were still more than 80% of older children had a 25(OH)D level at < 75 nmol/L (Figure 3). But among young children, the levels of 25(OH)D in winter were much higher than in spring, and the prevalence of 25(OH)D levels at < 75 nmol/L and < 50 nmol/L in winter were much lower than that in spring (Figures 2 and 3).

## Discussion

The present study is the first one to investigate vitamin D status among the pediatric population in Hangzhou, Zhejiang province, the southeast China. Our data demonstrate that the serum 25-hydroxyvitamin D levels among pediatric population decreased when the children became older, and the prevalence of vitamin D deficiency increased at the same time. The mean level of 25(OH) D among infants was much higher than that of any other stages. This is mainly because of the recommendation by the Pediatric Branch of Chinese Medical Association that all children receive 400 IU/day of vitamin D from two weeks after born until they are 2 years old[22]. According to this recommendation, many of the children's health care providers will give parents advices about taking vitamin D supplementation in children's first two years of life. And there are many kinds of oral supplementations containing vitamins D and A for infants, made in aqua or oil drops and can be purchased in pharmacies in Hangzhou. So the parents are easy to get these kinds of supplementations for their infants. Furthermore the parents may will provide vitamin D fortified formula to their young children especially younger than 2 years according to the feeding recommendations for infants and toddlers [23].

But as to the children older than 2 years, we don't have any formal recommendations on vitamin D supplementations. Fewer and fewer children take vitamin D supplements when they grow older than 2 years. This may be one of the causes of the finding in the present study that the mean serum 25(OH)D levels of the children aged 2-16 years were much lower than that of infants, and the prevalence of vitamin D deficiency among children older than 2 years was much higher than that of infants.

When compared with American children, our children had much higher prevalence of vitamin D level at < 50 nmol/L in most of stages, 2-5 years (22% vs 14%), 6-11 years (40% vs 20%) [17] and adolescents (46% vs 28.8%)[24], although the prevalence of 25(OH)D < 25 nmol/L among our children was similar to that of American children. If the threshold is changed to < 75 nmol/L, almost all children aged 6-16 years had low levels (88.3% among children aged 6-11 years and 89.6% among adolescents) and most children aged 2-5 years did as well (68.6%).

Hangzhou is located in the southeast of China at northern latitude 30°. The solar exposure in Hangzhou is more ample than in northern areas. It is reported that the prevalence of vitamin D deficiency among adolescent girls in Beijing was 89% [25], much higher than that of our adolescent girls (52%). In Shanxi province, another northern area of China, the prevalence of 25(OH)D level < 50 nmol/L among children aged 12-24 months was 84.3% in spring and 8.1% in autumn[26,27], much higher than that of our infants (7.7% in spring and 3.4% in autumn).

Given the concerns for vitamin D deficiency in older children and adolescents, in November 2008 the American Academy of Pediatrics released a new recommendation that all children receive 400 IU/day of vitamin D from their first days of life through adolescence [28]. So we suggest that the recommendation on vitamin D supplementation for Chinese pediatric population should be revised to include older children and adolescents now that the low 25(OH)D status of Chinese children are more severe than that of American children.

In the present study, we also analyzed the vitamin D status in different seasons. We found that the mean levels of serum 25(OH)D among children aged 6-16 years changed according to seasons. The levels were very low in winter, increased gradually in spring and summer, and reached the peaks in autumn. This tendency was similar to that of American adolescents [24]. But there were strange findings in our study, that the mean serum 25(OH)D levels among children aged 0-5 years in winter were as high as that in summer or autumn and significantly higher than that in spring. The possible reason is that the vitamin D supplementation for young children may be emphasized in winter when the sunlight is weak and the children will be dressed in many layers when they go outdoors. We did not find any significant differences between boys and girls at any stage. But the mean serum 25(OH)D level among adolescent girls was lower than that of boys, *P *= 0.05. This significant difference might be hidden by some reasons such as the uneven distribution of subjects between genders, now that it was reported that the serum 25(OH)D level was lower among American adolescent girls than that of boys[29]. The reason need to be explored in the future.

Although the subjects were not sampled from the whole pediatric population, they participated in the present study just for health examination with no medical status that might affect study results. Many of them took the examination before entered in kindergarten or school. Furthermore, the number of the sample was so large and the range of ages was also very big. So to some extent, the sample could represent the pediatric population of Hangzhou.

But there are still some limitations in the present study. The subjects were not sampled from the whole pediatric population in Hangzhou, and other possible relating factors of vitamin D status including intake of supplements, children's BMI and the time of physical activities were not collected. A further study based on subjects sampled on a population basis would be carried out and the possible relating factors of vitamin D status should be investigated. Furthermore, the optimal vitamin D level among children should to be certificated by more studies, now that adequate amount of vitamin D in all human populations for sustaining both innate and acquired immunity against infection is very important [8]. While at the same time, some authors warned that the prevalence of vitamin D deficiency was over estimated and the evidence of the role of vitamin D for extraskeletal outcomes was inconsistent and inconclusive and need to be assessed by more randomized clinical trials [30,31].

## Conclusions

The prevalence of vitamin D deficiency and insufficiency among children in Hangzhou Zhejiang province is high, especially among children aged 6-16 years. We suggest that the recommendation for vitamin D supplementation in Chinese children should be extended to adolescence.

## Competing interests

The authors declare that they have no competing interests.

## Authors' contributions

ZwZ (the first author) and ZyZ (the corresponding author) were responsible for the conception and design of the study. LC carried out the 25-hydroxyvitamin D measurement. ZwZ and WC performed the data analysis and drafted the manuscript. All authors participated in interpretation of the findings. ZyZ and JS revised and commented on the draft and all authors read and approved the final manuscript. All authors confirm that none of the content in the paper has been published or is under consideration for publication elsewhere.

## Pre-publication history

The pre-publication history for this paper can be accessed here:

http://www.biomedcentral.com/1471-2458/12/126/prepub
